# Genetic Characterization of a *bla*_VIM–24_-Carrying IncP-7β Plasmid p1160-VIM and a *bla*_VIM–4_-Harboring Integrative and Conjugative Element Tn*6413* From Clinical *Pseudomonas aeruginosa*

**DOI:** 10.3389/fmicb.2019.00213

**Published:** 2019-02-26

**Authors:** Lijun Zeng, Zhe Zhan, Lingfei Hu, Xiaoyuan Jiang, Yanjun Zhang, Jiao Feng, Bo Gao, Yuee Zhao, Wenhui Yang, Huiying Yang, Zhe Yin, Dongsheng Zhou

**Affiliations:** ^1^State Key Laboratory of Pathogen and Biosecurity, Beijing Institute of Microbiology and Epidemiology, Beijing, China; ^2^Department of Clinical Laboratory, The 307th Hospital of the Chinese People’s Liberation Army, Beijing, China; ^3^Zhejiang Provincial Center for Disease Control and Prevention, Hangzhou, China

**Keywords:** IncP-7 plasmid, unit transposon, integrative and conjugative element, *bla*_VIM_, *Pseudomonas aeruginosa*

## Abstract

This study presents three novel integrons In1394, In1395, and In1443, three novel unit transposons Tn*6392*, Tn*6393*, and Tn*6403*, one novel conjugative element (ICE) Tn*6413*, and the first sequenced IncP-7 resistance plasmid p1160-VIM from clinical *Pseudomonas aeruginosa*. Detailed sequence comparison of p1160-VIM (carrying Tn*6392* and Tn*6393*) and Tn*6413* (carrying Tn*6403*) with related elements were performed. Tn*6392*, Tn*6393*, and Tn*6403* were generated from integration of In1394 (carrying *bla*_VIM–24_), In1395 and In1443 (carrying *bla*_VIM–4_) into prototype Tn*3*-family unit transposons Tn*5563*, Tn*1403*, and Tn*6346*, respectively. To the best of our knowledge, this is the first report of a *bla*_VIM–24_-carrying *P. aeruginosa* isolate.

## Introduction

Plasmids of thirteen incompatibility groups in *Pseudomonas* (IncP-1 to IncP-7 and IncP-9 to IncP-14) have been recognized, varying in genetic structure, size and host range. IncP-7 plasmids, with a narrow host range, are of particular interest in environmental biodegradative potentials. Most sequenced members of this group, such as pCAR1 (Maeda et al., 2003), pND6_1 (Li et al., 2004), pWW53 (Pickup and Williams, 1985), pDK1 (Kunz and Chapman, 1981), and pHE24 (Supplementary Table S1), belong to toluene catabolic or degradation plasmids (D-plasmids) rather than resistance plasmids (R-plasmids).

Integrative and conjugative elements (ICEs), also known as conjugative transposons, are typically found integrated into host bacterial chromosomes and encode integrase (Int), excisionase (Xis) and type IV secretion system responsible for integration, excision, interbacterial transfer, respectively. ICEs confer antibiotic resistance (such as Tn*916*) (Franke and Clewell, 1981), heavy metal resistance (such as R391) (Peters et al., 1991), and carbon utilization (such as ICEclc) (Gaillard et al., 2006).

Verona integron-encoded metallo-β-lactamase (VIM) is one of the most predominant families among class B carbapenemases and can hydrolyze nearly all β-lactams including carbapenems, except aztreonam (Queenan and Bush, 2007). This study dealt with a detailed genetic characterization of a novel *bla*_VIM–24_-carrying IncP-7β plasmid p1160-VIM and a novel *bla*_VIM–4_-carrying ICE Tn*6413* recovered from two different clinical *P. aeruginosa* isolates.

## Materials and Methods

### Bacterial Isolates

*Pseudomonas aeruginosa* 1160 was isolated in 2015 from a sputum specimen of an elderly patient in a teaching hospital in Hebei Province, China. *P. aeruginosa* 6762 was recovered in 2016 from a sputum specimen of an elderly patient in a public hospital in Lanzhou Province, China. Bacterial species was identified by 16S rRNA gene sequencing and PCR detection of *P. aeruginosa*-specific *oafA* gene (Choi et al., 2013).

### Conjugal Transfer

Conjugal transfer experiments were carried out with rifampin-resistant *P. aeruginosa* PAO1 used as recipients and each of the *bla*_VIM_-positive 1160 or 6762 isolate as donor. Three milliliters of overnight cultures of each of donor and recipient bacteria were mixed together, harvested and resuspended in 80 μl of Brain Heart Infusion (BHI) broth (BD Biosciences). The mixture was spotted on a 1 cm^2^ hydrophilic nylon membrane filter with a 0.45 μm pore size (Millipore) that was placed on BHI agar (BD Biosciences) plate and then incubated for mating at 30°C for 12 to 18 h. Bacteria were washed from filter membrane and spread on Muller-Hinton (MH) agar (BD Biosciences) plates containing 1000 μg/ml rifampin together with 2 μg/ml meropenem for selecting an *P. aeruginosa* transconjugant carrying *bla*_VIM_.

### Sequencing and Annotation

The genomic DNA of strain 6762 or the plasmid DNA of strain 1160 was isolated using an UltraClean Microbial Kit or a Large Construct Kit (Qiagen, NW, Germany), respectively, and then sequenced from a mate-pair library with average insert size of 5 kb (ranged from 2 to 10 kb) using a MiSeq sequencer (Illumina, CA, United States). DNA contigs were assembled based on their contig coverages using Newbler 2.6 (Nederbragt, 2014). Open reading frames and pseudogenes were predicted using RAST 2.0 (Brettin et al., 2015) combined with BLASTP/BLASTN (Boratyn et al., 2013) searches against the UniProtKB/Swiss-Prot database (Boutet et al., 2016) and the RefSeq database (O’Leary et al., 2016). Annotation of resistance genes, mobile elements, and other features was carried out using the online databases including CARD (Liang et al., 2017), ResFinder (Zankari et al., 2012), ISfinder (Siguier et al., 2006), INTEGRALL (Moura et al., 2009), and the Tn Number Registry (Roberts et al., 2008). Multiple and pairwise sequence comparisons were performed using MUSCLE 3.8.31 (Edgar, 2004) and BLASTN, respectively. Gene organization diagrams were drawn in Inkscape 0.48.1^1^.

### Phylogenetic Analysis

The nucleotide sequences of *repA* coding regions of indicative plasmids were aligned using MUSCLE 3.8.31 (Edgar, 2004). The unrooted neighbor-joining trees were generated from the aligned *repA* sequences using MEGA7 (Kumar et al., 2016), and evolutionary distances were estimated using the maximum composite likelihood method, with a bootstrap iteration of 1000.

### Phenotypic Assays

Activity of Ambler class A/B/D carbapenemases in bacterial cell extracts was determined by a modified CarbaNP test (Wei et al., 2016). Bacterial antimicrobial susceptibility was tested by BioMérieux VITEK 2 and interpreted as per the 2017 Clinical and Laboratory Standards Institute (CLSI) guidelines (Wayne, 2017).

### Nucleotide Sequence Accession Numbers

The sequence of p1160-VIM and that of the 6762 chromosome were submitted to GenBank under accession numbers MF144194 and CP030075, respectively.

## Results and Discussion

### Overview of Sequenced p1160-VIM and Tn*6413*

Two *bla*_VIM_-positive *P. aeruginosa* isolates, designated 1160 and 6762, were subjected to high-throughput genome sequencing. The 1160 isolate harbored a *bla*_VIM−24_-carrying plasmid p1160-VIM, which had a circular DNA sequence of 205.4 kb in length, with an average G+C content of 56.3%. p1160-VIM belonged to the IncP-7 group because it had a IncP-7 *repA* gene responsible for plasmid replication initiation.

A 114.1-kb *bla*_VIM–4_-harboring ICE Tn*6413* was found to integrate into tRNA*^Gly^* gene in the 6762 chromosome. The modular structure of each of p1160-VIM and Tn*6413* was divided into the backbone (responsible for replication, maintenance and conjugal transfer) and separate accessory modules (defined as acquired DNA regions associated with mobile elements) integrated at different sites of the backbone (Supplementary Figures S1, S2 and Table 1).

**Table 1:** Major features of plasmids and ICEs analyzed.

Category	Plasmids			Chromosomally integrated ICEs			
	pCAR1	pDK1	p1160-VIM	Tn*6413*	Tn*6533*	Tn*6534*	Tn*6417*
Accession number	AB088420	AB434906	MF144194	CP030075	AP014651	KX196168	CP013993
Group	IncP-7α	IncP-7β	IncP-7β	Tn*6417*	Tn*6417*	Tn*6417*	Tn*6417*
Reference of the relevant group	Yes	Yes					Yes
Total length (bp)	199,035	128,921	205,426	114,067	109,026	118,715	108,186
Total number of ORFs	217	117	237	157	112	104	107
Mean G+C content, %	56.3	56	56.2	60.5	61.3	61.3	61.3
Length of the backbone (bp)	115,716	76,947	135,455	84,038	84,181	83,215	85,992
Accessory modules	Tn*4676*, IS*pa73*, a Tn*3*-family transposon remnant, IS*Pre3*, and IS*Pre4*	Tn*4662*, Tn*4663*, IS*1162*, and IS*pa81*	Tn*6392*^$^, Tn*6393*^$^, IS*pa75*, IS*pa79*, IS*pa80*, IS*pa81*, IS*pa83*, and IS*Pre2*	Tn*6403*^$^	Tn*6531*^$^	Tn*6530*^$^	Tn*6532*^$^
Host bacterium	*P. resinovorans* CA10	*P. putida* HS1	*P. aeruginosa* 1160	*P. aeruginosa* 6762	*P. aeruginosa* NCGM257	*P. aeruginosa* RI_IH-2	*P. aeruginosa* DHS01
Nucleotide positions in the chromosome	–	–	–	337873.. 451939	5233626.. 5342651	1..118715	5365108.. 5473293

*p1160-VIM and Tn6413 were sequenced this work, and all the other elements analyzed were derived from GenBank. $, carrying resistance genes.*

p1160-VIM could be transferred from the 1160 isolate into *P. aeruginosa* PAO1 through conjugation, generating the transconjugant 1160-VIM-PAO1. The self-transmissible nature of p1160-VIM was consistent with the presence of complete conjugal transfer regions in this plasmid. Strains 1160 and 1160-VIM-PAO1 had class B carbapenemase activity, and they were resistant to cefuroxime, ceftazidime, ceftriaxone and cefepime (with minimal inhibitory concentration values ≥ 64), and imipenem and meropenem (with minimal inhibitory concentration values ≥ 4), which were resulted from production of VIM enzymes in these strains. Repeated conjugation attempts failed to transfer Tn*6413* from the 6762 isolate to PAO1.

### Subgrouping of IncP-7 Plasmids Including p1160-VIM

**FIGURE 1:**
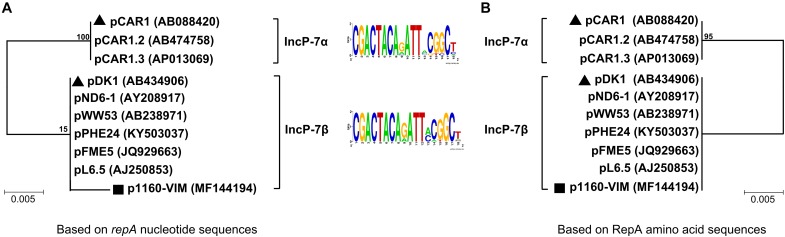
Neighbor-joining phylogenetic trees of sequenced IncP-7 plasmids. Phylogenetic trees are constructed based on *repA* nucleotide **(A)** and amino acid sequences **(B)**, respectively. Degree of support (percentage) for each cluster of associated taxa, as determined by bootstrap analysis, is shown next to each branch. Triangles indicate IncP-7α and IncP-7β reference plasmids, while squares denote p1160-VIM sequenced in this work.

A group of ten completely or partially sequenced plasmids (Supplementary Table S1; including p1160-VIM) with IncP-7 *repA* genes (≥ 95% nucleotide identity to that of p1160-VIM), were collected, and two phylogenetic trees (Figure 1) were constructed based on *repA* nucleotide and amino acid sequences, respectively. These ten plasmids could be divided into two separately clustering subgroups designated IncP-7α and IncP-7β. As shown by pairwise comparison of *repA* nucleotide sequences, plasmids within each of these two subgroups showed ≥ 99% nucleotide identity, while plasmids from these two different subgroups displayed ≤ 96% nucleotide identity (Supplementary Table S2a). Considerable genetic diversity was found between the *repA* genes of IncP-7α and IncP-7β, representing two separated lineages.

Predicted iterons (RepA-binding sites) were found within the *oriV* region downstream of *repA*, and plasmids from both subgroups shared a conserved iteron motif and an identical iteron copy number (Figure 1 and Supplementary Table S1).

pCAR1 (Maeda et al., 2003) and pDK1 (Kunz and Chapman, 1981) were identified as IncP-7α and IncP-7β reference plasmids, respectively, because they were the first sequenced plasmids harboring complete conjugal transfer regions. In the phylogenetic tree based on nucleotide sequences, p1160-VIM displayed a long branch, which resulted from presence of five single nucleotide polymorphisms (SNPs) in p1160-VIM, while all other plasmids had identical *repA* sequences (Supplementary Figure S3). Notably, these five SNPs did not lead to mutations of RepA amino acid sequences.

### Comparison of p1160-VIM With pCAR1 and pDK1

**FIGURE 2:**
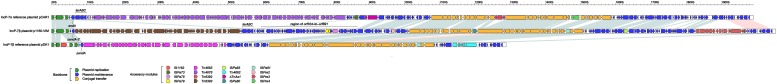
Linear comparison of p1160-VIM with related plasmids. A linear comparison is carried out for complete DNA sequences of pCAR1 (accession number AB088420), p1160-VIM (this study), and pDK1 (accession number AB434906). Genes are denoted by arrows. Genes, mobile elements and other features are colored based on function classification. Blue shading denotes regions of homology (> 95% nucleotide identity), and pink shading denotes regions of homology (nucleotide identity between 90 and 95%).

pCAR1, pDK1 and p1160-VIM were included in a genomic comparison. These three plasmids had > 92% nucleotide identity across > 52% of their backbone sequences (Supplementary Table S2b), and their conserved backbone was composed of gene or gene loci responsible for replication initiation (*repA*), partitioning (*parABCW*), and conjugal transfer (*rlx*, *cpl*, *tivF3*, and *tivF6*). There were three major modular differences within their backbones (Figure 2): (i) a *terABC* region could be found in p1160-VIM and pCAR1 rather than pDK1; (ii) a 23.9-kb *orf324–*to*–orf891* region was found in only p1160-VIM; and (iii) the *endA* gene was intact in pCAR1 but was interrupted or truncated in p1160-VIM and pDK1. All these modular differences were resulted from integration of relevant accessory modules.

pCAR1, pDK1, and p1160-VIM carried totally different profiles of accessory modules (Table 1), which were composed of 10 distinct IS elements (IS*Pre2*, IS*Pre3*, IS*Pre4*, IS*1162*, IS*pa73*, IS*pa75*, IS*pa79*, IS*pa80*, IS*pa81*, and IS*pa83*), 5 different intact Tn*3*-family unit transposons (Tn*4676* from pCAR1, Tn*4662*, and Tn*4663* from pDK1, and Tn*6392* and Tn*6393* from p1160-VIM; a typical unit transposon encodes a transposase and a site-specific recombinase or resolvase as core transposition determinants, and also carries one or several accessory genes), and one Tn*3*-family transposon remnant. Only Tn*6392* and Tn*6393* of the above accessory modules (Table 2). Tn*4676* (Supplementary Figure S4a) carried core transposition genes (*tnpAC* and *tnpST*) genetically related to Tn*4651* (Maeda et al., 2003), and also an *ant* (two-component anthranilate 1,2-dioxygenase) operon (Urata et al., 2004) interrupted by insertion of IS*Pre1* and a *car* (carbazole/dioxin degradation) operon (Nojiri et al., 2001). Tn*4662* encoded a RelBE toxin-antitoxin system involved in plasmid maintenance. Tn*4663* (Supplementary Figure S4b) was derived from Tn*4659* (Yano et al., 2007) and harbored a toluene-catabolic *xyl* gene cluster (Yano et al., 2010).

**Table 2:** Drug resistance genes in mobile elements sequenced this study.

Mobile element	Resistance marker	Resistance phenotype	Nucleotide position	Region located
p1160-VIM	*strAB*	Aminoglycoside resistance	8845..10484	Tn*6393*
	*sul1*	Sulphonamide resistance	18181..19020 40901..41740	
	*qacED1*	Quaternary ammonium compound resistance	19014..19361 41734..42081	
	*folA*	Trimethoprim resistance	19489..20022	
	*qnrVC*	Quinolone resistance	26051..26707	
	*mph(E)*	Macrolide resistance	34491..35375	
	*msr(E)*	Macrolide resistance	35431..36906	
	*aadA1a*	Streptomycin resistance	43627..44418	
	*catB3q*	Chloramphenicol resistance	46142..46774	
	*ereA1c*	Erythromycin resistance	46884..48104	
	*aacA4*	Aminoglycoside resistance	188445..188963 189993..190511	Tn*6392*
	*bla*_VIM–24_	Carbapenem resistance	189102..189902	
	*mer*	Mercuric resistance	195914..197025	
Tn*6413*	*aadA2*	Streptomycin resistance	398558..399337	—
	*qacED1*	Quaternary ammonium compound resistance	399501..399848 411418..411765	
	*sul1*	Sulphonamide resistance	399842..400681 411759..412598	
	*msr(E)*	Macrolide resistance	402951..404426	
	*mph(E)*	Macrolide resistance	404482..405366	
	*bla*_VIM–4_	Carbapenem resistance	409112..409996	
	*aacA7*	Aminoglycoside resistance	410090..410548	
	*aacA4*	Aminoglycoside resistance	410731..411249	
	*mer*	Mercuric resistance	414543..418060	

### Comparison of Tn*6392* With Tn*5563*

**FIGURE 3:**
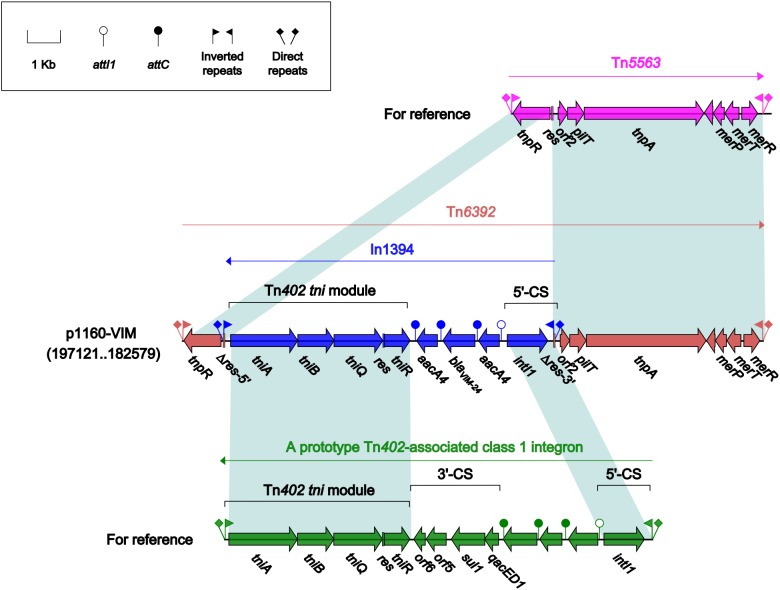
Organization of Tn*6392* and comparison to related regions. Genes are denoted by arrows. Genes, mobile elements and other features are colored based on their functional classification. Shading denotes regions of homology (nucleotide identity > 95%). Numbers in brackets indicate nucleotide positions within corresponding plasmid sequences. Accession number of Tn*5563* for reference is U88088.

Tn*6392* (Figure 3) from p1160-VIM was a novel derivative of Tn*5563*, which was originally characterized in *P. alcaligenes* and had the structure IRL (inverted repeat left)–*tnpR* (resolvase)–*res* (resolution site)–*orf2* (hypothetical protein)–*pliT* (*pilT* domain-containing protein)–*tnpA* (transposase)–*mer* (mercuric resistance gene locus)–IRR (inverted repeat right), bracketed by 5-bp or 7-bp direct repeats (DRs; target site duplication signals) at both ends (Yeo et al., 1998). Tn*6392* differed from Tn*5563* by insertion of a novel class 1 integron In1394 into *res*. The prototype Tn*402*-associated class 1 integron was typically organized as IRi (inverted repeat at the integrase end), 5’-CS [5’-conserved segment: *intI1* (integrase)-*attI1* (a specific recombination site)], GCA (gene cassette array), 3’-CS [3’-conserved segment: *qacED1–sul1–orf5–orf6*], a Tn*402 tni* module [*tniA* (transposase)*–tniB* (ATP-binding protein)*–tniQ* (transposition auxiliary protein)*–res–tniR* (serine resolvase)], and IRt (inverted repeat at the *tni* end) (Gillings et al., 2008). In1394, bracketed by 5 bp DRs at both ends, contained all the above core integron structures except 3’-CS. The GCA of In1394 consisted of a *bla*_VIM–24_ gene and two copies of *aacA4*.

### Comparison of Tn*6393* With Tn*1403*

**FIGURE 4:**
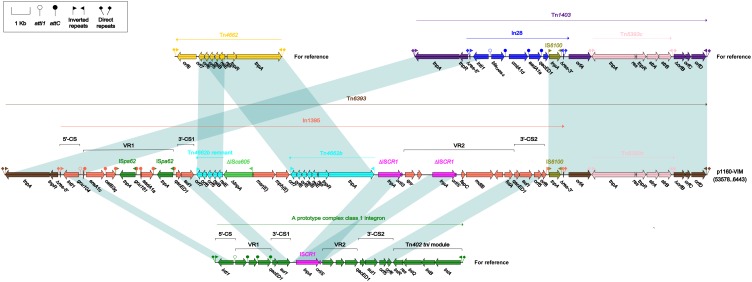
Organization of Tn*6393* and comparison to related regions. Genes are denoted by arrows. Genes, mobile elements and other features are colored based on their functional classification. Shading denotes regions of homology (nucleotide identity > 95%). Numbers in brackets indicate nucleotide positions within corresponding plasmid sequences. Accession number of Tn*1403* for reference is AF313472.

Tn*6393* (Figure 4) was a novel derivative from Tn*1403* after insertion of a novel class 1 integron In1395 instead of In28 at the same position within *res*. Tn*1403* was initially identified in *P. aeruginosa* and displayed a backbone structure IRL–*tnpAR*–*res*–*sup*–*uspA*–*dksA*–*yjiK*–IRR, with integration of accessory modules In28 and Tn*5393c* into *res* and *dksA*, respectively (Stokes et al., 2007). In1395 belonged to complex class 1 integron, which was typically organized as IRi*–*5’-CS*–*VR1 (variable region 1)*–*3’-CS1 (the first copy of 3’-CS1: *qacED1–sul1*)*–*IS*CR1* (comment region)–VR2 (variable region 2)–3’-CS2 (a second 3’-CS: *qacED1–sul1*–*orf5*–*orf6*)*–tni*–IRt. In1395, bracketed by 5-bp DRs at both ends, was composed of IRi, 5’-CS, VR1 [GCA: *gcu104*–*aacA1*–*catB3q*:IS*pa62*– *gcu161*–*ereA1c*:IS*pa62*], 3’-CS1, IS*CR1* (further interrupted by ΔTn*4662b–*ΔIS*cs605–msr*(E)*–mph*(E)*–*Tn*4662b*), VR2 [containing *qnr*, ΔIS*CR1*, *folA* and other genes], 3’-CS2, IS*6100* (replacing *tni*) and IRt.

### Comparison of Tn*6413* With Tn*6534*, Tn*6533*, and Tn*6417*

**FIGURE 5:**
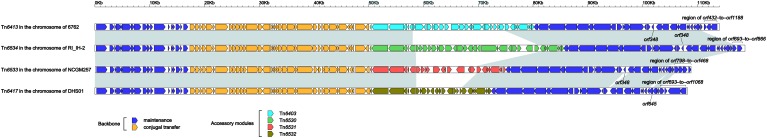
Linear comparison of Tn*6413* with related ICEs. A linear comparison is carried out for DNA sequences of Tn*6413* (this study), Tn*6534* (accession number KX196168), Tn*6533* (accession number AP014651), and Tn*6417* (accession number CP013993). Genes are denoted by arrows. Genes, mobile elements and other features are colored based on function classification. Shading denotes regions of homology (> 95% nucleotide identity).

**FIGURE 6:**
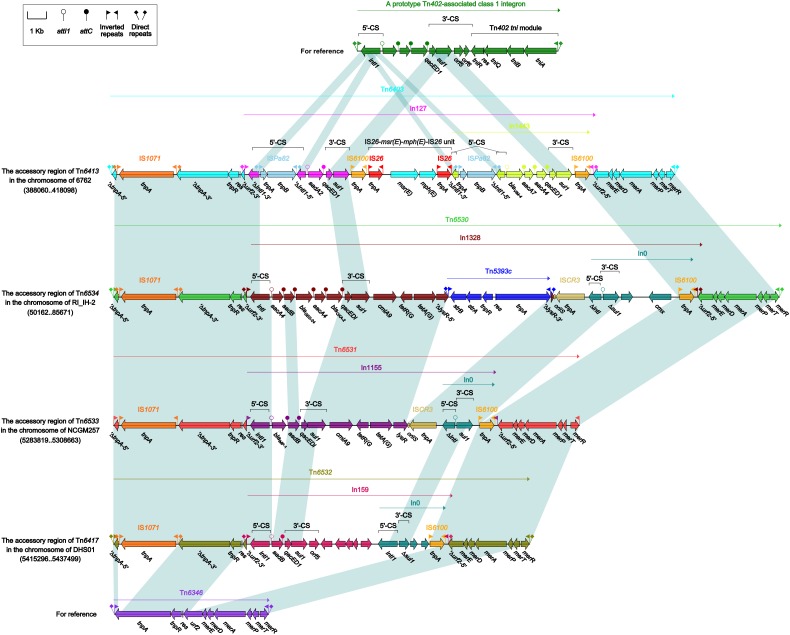
Organization of Tn*6403* and comparison with related regions. Genes are denoted by arrows. Genes, mobile elements and other features are colored based on their functional classification. Shading denotes regions of homology (nucleotide identity > 95%). Numbers in brackets indicate nucleotide positions within corresponding chromosome sequences. Accession number of Tn*6346* for reference is EU696790.

Tn*6413* (Supplementary Figure S2) was a novel ICE that could be divided into a single 30-kb accessory module Tn*6403* (Figure 6) and the remaining backbone regions. Tn*6413* belonged to a collection of 31 ICE or ICE-like sequences (Supplementary Table S3, including Tn*6417*, Tn*6534*, and Tn*6533*) with > 95% nucleotide identity across > 59% of Tn*6413* backbone. Tn*6417* was the first sequenced one and identified as the reference of these 31 Tn*6417*-family ICE sequences. A genomic comparison (Figure 5) was subjected to Tn*6413*, Tn*6534*, Tn*6533*, and Tn*6417* because they shared mostly highly similar backbones with 99% nucleotide identity and > 94% query coverage. These four Tn*6417*-family ICEs, which genetically differed from the two existing ICE families in *P. aeruginosa* (Kung et al., 2010) shared conserved DNA processing and conjugation genes. Three major modular differences were fund within the backbones of these four ICEs: (i) presence of *orf348* in only Tn*6417*; (ii) presence of *orf645* in only Tn*6417*; and iii) 3’-terminal regions (*orf432–*to*–orf1188*, *orf693*–to*–orf866*, *orf798*–to*–orf468*, and *orf693*–to*–orf1068* from Tn*6413*, Tn*6534*, Tn*6533* and Tn*6417*, respectively) differed from one another.

Each of these four Tn*6417*-family ICEs carried a single accessory module: Tn*6403*, Tn*6531*, Tn*6530*, and Tn*6532* (Figure 6) from Tn*6413*, Tn*6533*, Tn*6534*, and Tn*6417*, respectively; all these accessory modules were integrated at the same site of the ICE backbones and identified as Tn*6346* derivatives. The Tn*3*-family unit transposon Tn*6346*, originally found in heavy metal-tolerant *Achromobacter* spp., was a hybrid of the core transposition module *tnpAR*–*res* of Tn*5051* and the *mer* region of Tn*501* (Ng et al., 2009). Tn*6403*, Tn*6531*, Tn*6530*, and Tn*6532* differed from Tn*6346* by (i) interruption of original *tnpA*_Tn_*_6346_* due to insertion of IS*1071*, and (ii) insertion of four different class 1 integrons at the same position within the *urf2* gene of *mer*. Tn*6403*, Tn*6530* and Tn*6532*, rather than Tn*6531*, were bracketed by 5-bp DRs.

In127, In1328, In1155, and In159 found in Tn*6403*, Tn*6531*, Tn*6530*, and Tn*6532*, respectively, were intact integrons because all of them had paired terminal 25-bp repeats. Except In1155, all the other three were bracketed by 5-bp DRs. Notably, these integrons captured additional elements beside GCAs: IS*26*–*msr*(E)–*mph*(E)–IS*26* unit and a novel *bla*_VIM–4_-carrying class 1 integron In1443, *cmlA9*–*tetRA*(G)–Tn*5393c*–IS*CR3* and *cmx*-carrying In0, *cmlA9*–*tetRA*(G)–IS*CR3* and empty In0, and empty In0 in In127, In1328, In1155, and In159, respectively. In1443 was organized as IRi–5’-CS (interrupted by insertion of IS*Pa82*)–GCA (*bla*_VIM–4_–*aadA7*–*aadA4*)–Δ3’-CS–IS*6100* (replacing *tni*)–IRt.

## Conclusion

IncP-7 R-plasmids are not commonly found in natural isolates, and p1160-VIM represents the first fully sequenced IncP-7 R-plasmid. Based on *repA* sequences, IncP-7 plasmids can be further divided into two separately clustering subgroups IncP-7α and IncP-7β. The two novel *bla*_VIM_-carrying transposons Tn*6392* and Tn*6413*, which are integrated into the IncP-7β plasmid p1160-VIM and the *P. aeruginosa* chromosome, respectively, represent two different categories of transposons: Tn*3*-family unit transposon and Tn*6417*-family ICE. Tn*6392* and Tn*6413* contain novel class 1 integrons In1394 and In1443, which harbor the two GCAs *aacA4*–*bla*_VIM–24_–*aacA4* and *bla*_VIM–4-_*aadA7*–*aadA4*, respectively. The *bla*_VIM–24_ gene was initially discovered from a *Klebsiella pneumoniae* isolate in Colombia in 2011 (Montealegre et al., 2011). This study presents the first report of a *bla*_VIM–24_-carrying *P. aeruginosa* isolate and a *bla*_VIM_-carrying IncP-7 plasmid. Both p1160-VIM and Tn*6413* are conjugative (self-transmissible) mobile elements, promoting horizontal transfer of resistance genes carried. Presence of IRi/IRt and a complete *tni* module would ensure In1394 self-transferable, while replacement of *tni* by IS*6100* would impair mobility of In1443. Class 1 integrons (e.g., In1394 and In1443) could be integrated into a transposon (e.g., Tn*6392* and Tn*6413*) to restore or enhance their mobility.

## Ethics Statement

The use of human specimens and all related experimental protocols were approved by the Committee on Human Research of the First Affiliated Hospital of Hebei North University and that of the General Hospital of Xinjiang Military Region, and carried out in accordance with the approved guidelines. The research involving biohazards and all related procedures were approved by the Biosafety Committee of the Beijing Institute of Microbiology and Epidemiology.

## Author Contributions

DZ and ZY conceived the study and designed experimental procedures. LZ, ZZ, LH, XJ, and YJZ performed the experiments. LZ, ZZ, YJZ, JF, BG, YEZ, and WY analyzed the data. LZ, ZZ, and HY contributed reagents and materials. DZ, ZY, LZ, and ZZ wrote this manuscript.

## Conflict of Interest Statement

The authors declare that the research was conducted in the absence of any commercial or financial relationships that could be construed as a potential conflict of interest.

## Footnotes

1 https://inkscape.org/en/

## Supplementary Material

FIGURE S1 Plasmid schematic maps. Three plasmids pCAR1, pDK1 and p1160-VIM are included. Genes are denoted by arrows, and the backbone and accessory module regions are highlighted in black and color, respectively. Innermost circle presents GC-skew [(G–C)/(G+C)], with a window size of 500 bp and a step size of 20 bp. Next-to-innermost circle presents GC content.

FIGURE S2 ICE schematic maps. Four ICEs Tn*6413*, Tn*6534*, Tn*6533*, and Tn*6417* are included. Genes are denoted by arrows, and the backbone and accessory module regions are highlighted in black and color, respectively. Innermost circle presents GC-skew [(G–C)/(G+C)], with a window size of 500 bp and a step size of 20 bp. Next-to-innermost circle presents GC content.

FIGURE S3 Alignment of *repA* nucleotide sequences. Red-labeled nucleotides indicate SNP sites. Sequence of IncP-7β reference plasmid pDK1 was bolded.

FIGURE S4 Organization of Tn*4676* or Tn*4663* comparison with related regions. Genes are denoted by arrows. Genes, mobile elements and other features are colored based on their functional classification. Blue shading denotes regions of homology (nucleotide identity > 95%), and pink shading denotes regions of homology (average nucleotide identity 82%). Numbers in brackets indicate nucleotide positions within corresponding plasmid sequences. Accession number of Tn*4651* for reference is AJ344068.

TABLE S1 Collection of *repA* and iteron sequences of IncP-7 plasmids.

TABLE S2 Pairwise comparison of *repA* and backbone sequences.

TABLE S3 List of the *Tn6417*-related sequences.
