# Supplementary figures and images for: Genetic Characterization of a blaVIM–24-Carrying IncP-7β Plasmid p1160-VIM and a blaVIM–4-Harboring Integrative and Conjugative Element Tn6413 From Clinical Pseudomonas aeruginosa
Source: Front Microbiol. 2019 Feb 26;10:213. doi: 10.3389/fmicb.2019.00213 (PMC6399125; doi:10.3389/fmicb.2019.00213)

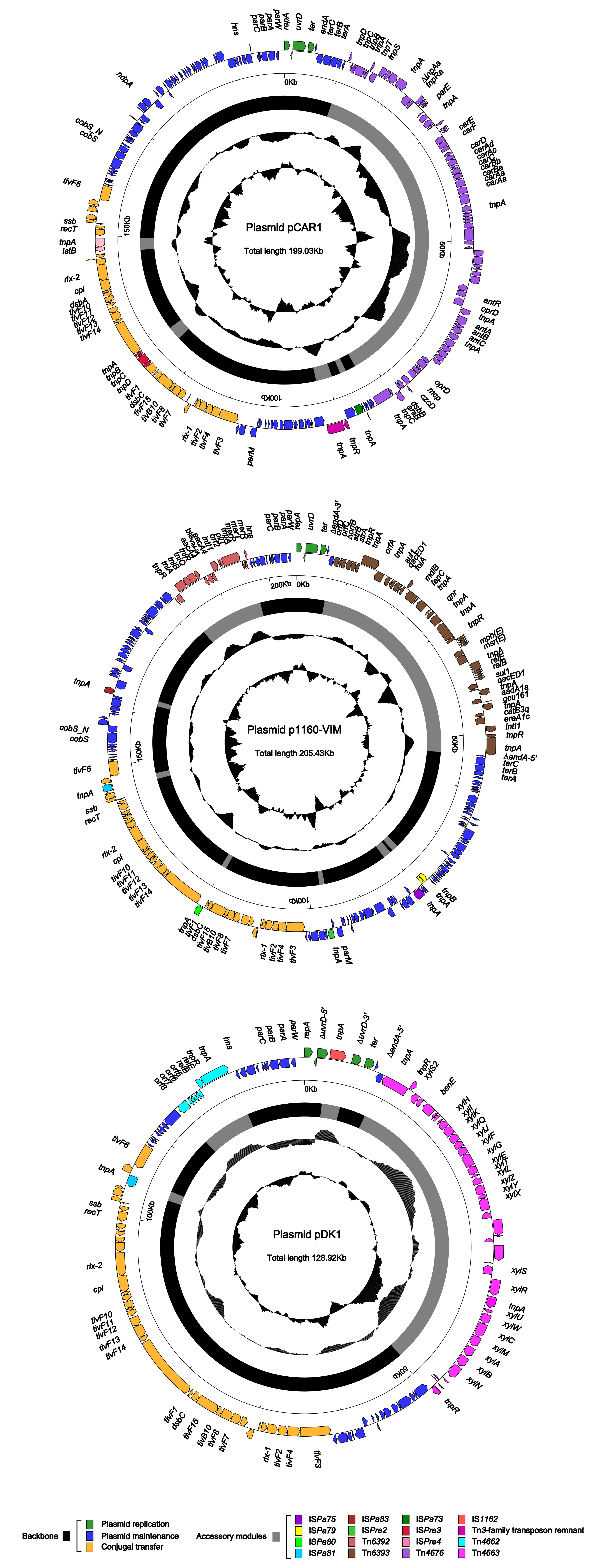

Supplement: FIGURE S1 — Plasmid schematic maps. Three plasmids pCAR1, pDK1 and p1160-VIM are included. Genes are denoted by arrows, and the backbone and accessory module regions are highlighted in black and color, respectively. Innermost circle presents GC-skew [(G–C)/(G+C)], with a window size of 500 bp and a step size of 20 bp. Next-to-innermost circle presents GC content. [file Image_1.tif]

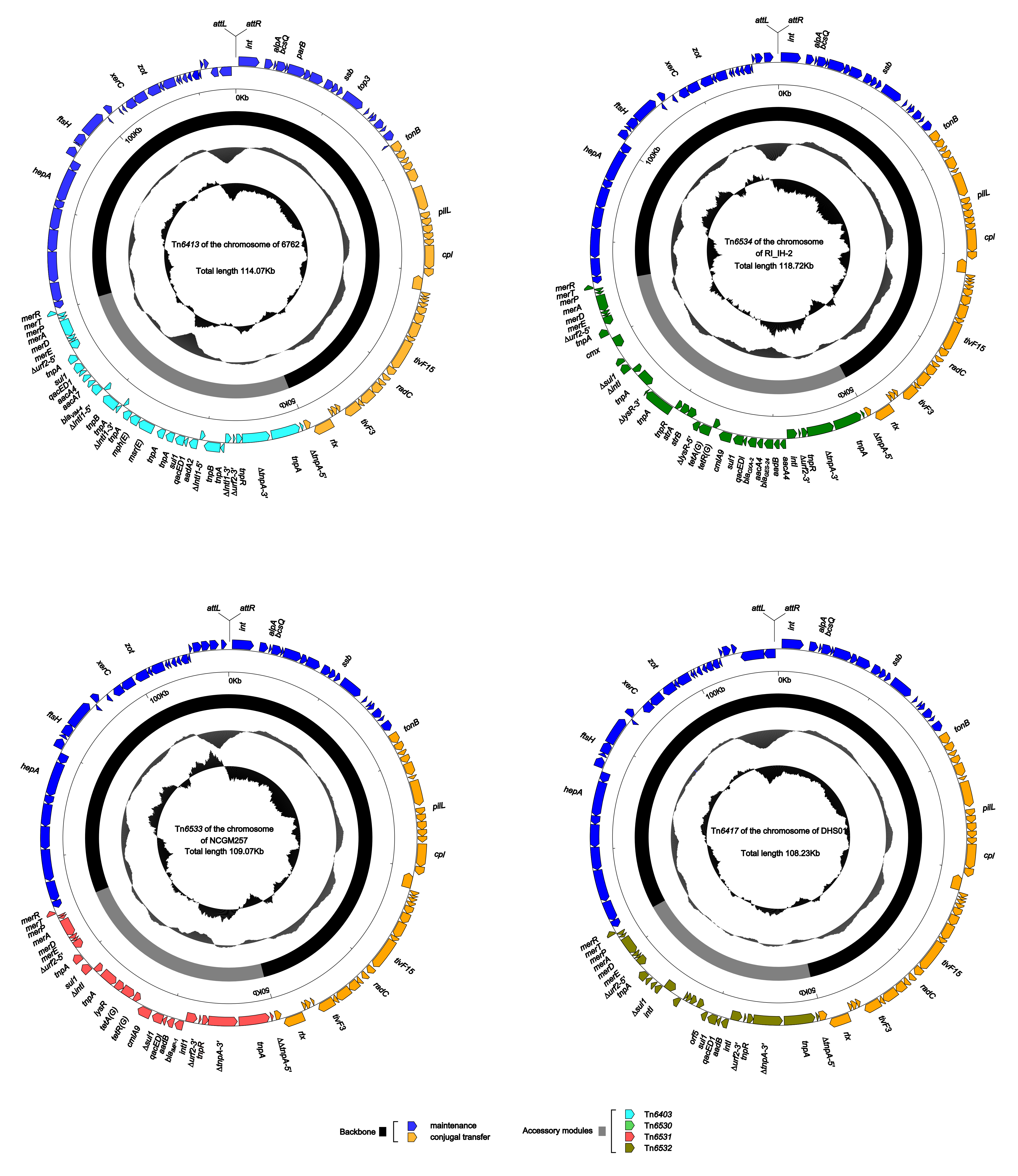

Supplement: FIGURE S2 — ICE schematic maps. Four ICEs Tn6413, Tn6534, Tn6533, and Tn6417 are included. Genes are denoted by arrows, and the backbone and accessory module regions are highlighted in black and color, respectively. Innermost circle presents GC-skew [(G–C)/(G+C)], with a window size of 500 bp and a step size of 20 bp. Next-to-innermost circle presents GC content. [file Image_2.tif]

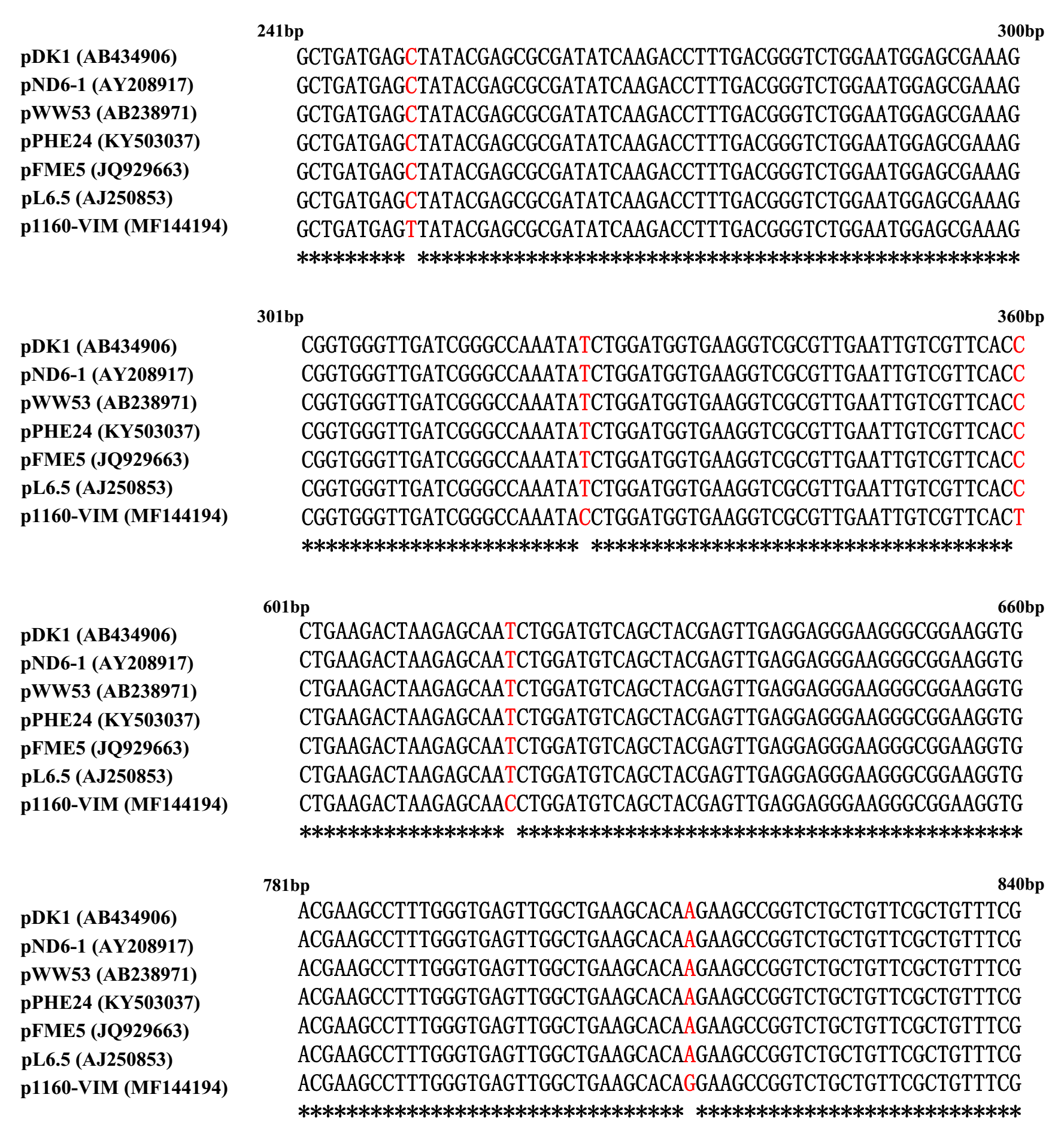

Supplement: FIGURE S3 — Alignment of repA nucleotide sequences. Red-labeled nucleotides indicate SNP sites. Sequence of IncP-7β reference plasmid pDK1 was bolded. [file Image_3.tif]

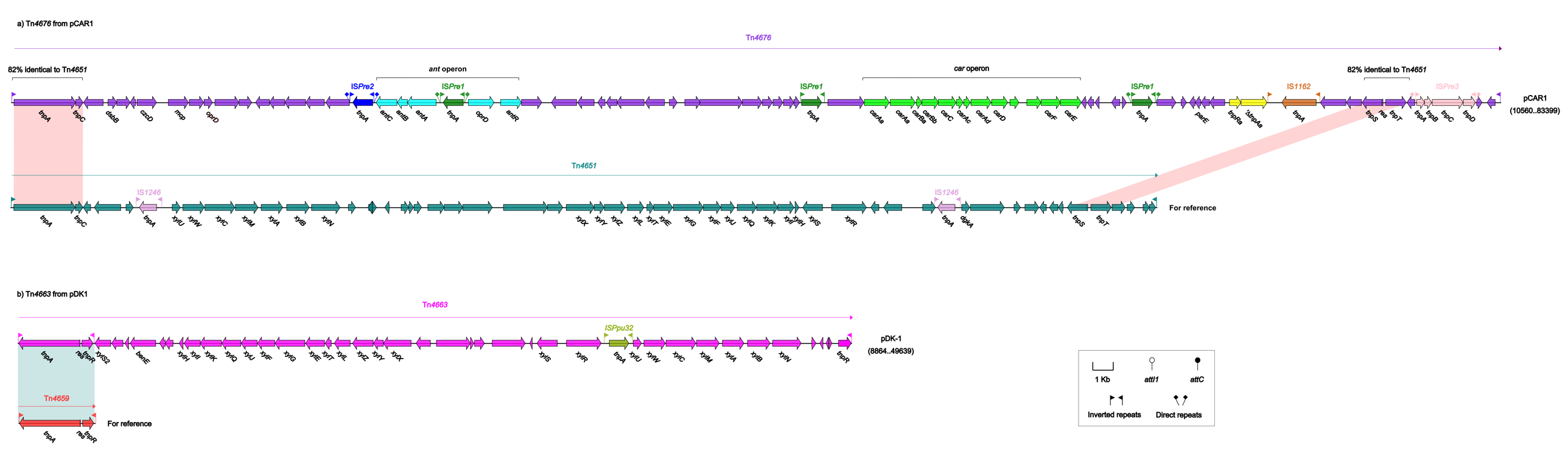

Supplement: FIGURE S4 — Organization of Tn4676 or Tn4663 comparison with related regions. Genes are denoted by arrows. Genes, mobile elements and other features are colored based on their functional classification. Blue shading denotes regions of homology (nucleotide identity > 95%), and pink shading denotes regions of homology (average nucleotide identity 82%). Numbers in brackets indicate nucleotide positions within corresponding plasmid sequences. Accession number of Tn4651 for reference is AJ344068. [file Image_4.tif]
